# Simple Acid Digestion Procedure for the Determination of Total Mercury in Plankton by Cold Vapor Atomic Fluorescence Spectroscopy

**DOI:** 10.3390/mps5020029

**Published:** 2022-03-25

**Authors:** João Pereira Santos, Lirie Mehmeti, Vera I. Slaveykova

**Affiliations:** Environmental Biogeochemistry and Ecotoxicology, Department F.-A. Forel for Environmental and Aquatic Sciences, School of Earth and Environment Sciences, Faculty of Sciences, University of Geneva, 1211 Geneva, Switzerland; lirie.mehmeti@unige.ch

**Keywords:** wet acid digestion, IAEA-450, BCR-414, plankton, microalgae, zooplankton, total mercury, CVAFS

## Abstract

Plankton, at the bottom of the food web, play a central role in the entry of mercury into the aquatic biota. To investigate their role in mercury uptake, reliable analytical procedures for Hg analysis are highly sought. Wet digestion procedures for determining total mercury in different biological matrices have been established since years, however only few studies focused on planktonic samples. In the present work, a simple and cost-effective wet digestion method was developed for the determination of total mercury in samples of small plankton material using a cold vapor atomic fluorescence spectroscopy (CVAFS). The optimization of the digestion method was achieved by using glass vessels with Teflon caps, low amount of acids (3 mL *w*/*w* 65% HNO_3_ or 3 mL 50% *v*/*v* HNO_3_), a constant temperature of 85 °C, the presence and absence of pre-ultrasound treatment, and a continuous digestion period (12 h). Certified reference materials IAEA-450 (unicellular alga Scenedesmus obliquus) and BRC-414 (plankton matrix) were used to optimize and validate the digestion method. The recovery efficiency of the proposed method for IAEA-450 and BCR-414 (3.1 mg and 21.5 mg) ranged between 94.1 ± 7.6% and 97.2 ± 4.6%. The method displayed a good recovery efficiency and precision for plankton matrices of low size. Thus, allowing better digestion of planktonic samples for mercury analysis using CVAFS techniques.

## 1. Introduction

Mercury is a pollutant of global concern that can affect both human and ecosystem health [1]. Despite the low environmental concentrations of mercury, the capacity of inorganic mercury (Hg(II)) to bioaccumulate and methylmercury to biomagnify throughout the aquatic food web [2,3,4] poses a great threat to human health primarily by seafood consumption. Mercury enters the aquatic food web mainly via accumulation by plankton at its basis [2,5]. Therefore, the phyto- and zoo-plankton play a crucial role in the Hg trophic transfer in aquatic environments [6]. Monitoring the accumulation of mercury in this particular group of organisms is thus of utmost importance when it comes to understanding the mercury bioavailability and entry into the food webs in the aquatic environment.

Nevertheless, the lack of standard methods adapted to phytoplankton sample preparation and subsequent analysis has resulted in inconsistencies in data reported by different laboratories, leading to variations in mercury recoveries [7]. There are two main approaches to determine total mercury from solid matrices. The first comprises the direct analysis of sample matrices performed by direct mercury analyzers (DMAs) that is based on sample combustion, concentration of mercury by amalgamation with gold, and cold vapor atomic absorption spectrometry (CVAAS) [8,9]. The second approach requires the decomposition of solid matrices for extraction of mercury [10], usually through wet digestion [11,12,13,14] (commonly with a mix of acids or bases for later thermal digestion) or microwave-assisted digestion [15,16,17,18] with or without a preliminary assisted ultrasound extraction [19] followed by its quantification using spectroanalytic techniques (e.g., cold vapor atomic fluorescence spectroscopy (CVAFS)). The vast majority of the wet digestion procedures are developed for soils and sediments [14,17,18,20], and biological samples (such as fish tissues, mussels) [1,10,14,17] ranging between 0.2 and 2 g of dry weight. Planktonic samples are usually available in low milligrams range, thus posing a problem with reproducible decomposition of the biological matrices due to the important influence of the sample’s heterogeneity. In addition, there is no widely accepted standard methodology to extract mercury from such biological matrices, though some studies have addressed these type of biological matrices [11,16,21] and taken into account the yielded low-biomass factor using microwave digestion method [16]. Depending on the type of the biological matrix, optimization of the sample digestion should be performed [22,23]. The main parameters usually optimized for the decomposition of biological matrices for mercury analysis are the type of digestion reagent, time of digestion, and temperature [7,12,13,14,17,24]. However, other factors, such as purity of reagents and use of trace metal clean material protocols, are very important, especially when working with metals such as mercury since the possibility of contamination increases greatly [25]. Using certified referenced material in the present study, we optimized the digestion methodology conditions to achieve good accuracy and precision of the extraction of mercury from the plankton samples available at the milligrams scale.

## 2. Material and Methods

### 2.1. Materials and Reagents

Certified reference materials (CRM) used for the method validation are unicellular microalga *Scenedesmus obliquus* (IAEA-450) and plankton material (BCR-414) from International Atomic Energy Agency and European Joint Research Centre, respectively.

All plasticware was pre-washed in 10% *v*/*v* HNO_3_ (EMSURE, Merck, Darmstadt, Germany), followed twice by a 10% *v*/*v* HCl acid bath (EMSURE, Merck, Darmstadt, Germany) for 1 h under sonication bath, thoroughly rinsed with ultrapure water (Milli-Q direct system, Merck, Darmstadt, Germany). All glassware was pre-washed in a 10% *v*/*v* HCl acid bath (EMSURE, Merck, Darmstadt, Germany) overnight, thoroughly rinsed with ultrapure water and then pyrolyzed at 550 °C for at least 1 h. Teflon caps used for both digestion and analysis were acquired at Brooks Rand Instruments (Seattle, WA, USA).

Inorganic mercury stock solution (0.0001% *w*/*v*) dissolved in HNO_3_ (2% *v*/*v*) was acquired by Brooks Rand Instruments (Seattle, WA, USA). Working solutions of Hg(II) were freshly prepared by diluting in ultrapure water appropriate aliquot of the mercury stock solution followed by the addition of 100 µL bromine monochloride (BrCl) to maintain mercury oxidation form as described on EPA Method 1631 [22]. BrCl reagent (2.6% *w*/*v*), Hydroxylamine HCl (NH_2_OH * HCl) reagent (30% *w*/*v*) with antifoaming and Stannous Chloride (20% *w*/*v*) in 10% *v*/*v* HCl were acquired at Brooks Rand Instruments (Seattle, WA, USA).

Reagents used for the sample digestion procedure consisted of HNO_3_ (65% *w*/*w*, for analysis (max. 0.005 ppm Hg) EMSURE^®^) or 50% *v*/*v* HNO_3_ diluted in ultrapure water (18.2 MΩ, Millipore, Darmstadt, Germany).

### 2.2. Sample Preparation and Digestion

**Figure 1:**
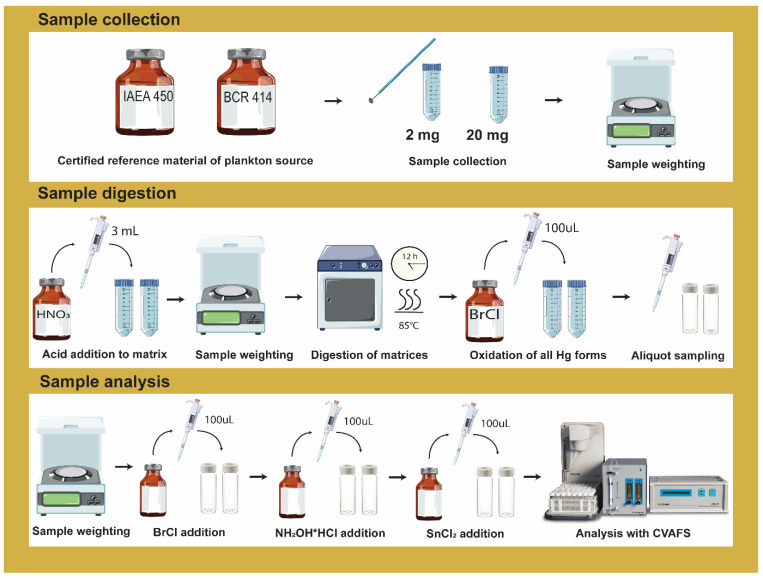
Illustrative scheme of the wet acid digestion procedure used on this study with the three stages well described: sample collection, sample digestion and sample analysis.

A preliminary screening with different conditions was evaluated to optimize the method conditions considering the available literature. The influence of acid concentration (50% *v*/*v* HNO_3_ and 65% *w*/*w* HNO_3_), period of digestion (12 h and 8 h), and the presence or absence of ultrasound treatment (10 min ultrasound bath prior to wet digestion) were explored (Table S1), as they seemed to be more prompt to achieve good accuracy and recovery efficiency. Results obtained from the digestion screening are displayed in Figure S1. From this screening, two conditions (conditions 1 and 2, Table S1) were selected for further optimization of the methodology. Two different CRM (IAEA-450 and BCR-414) were used on all procedures with two different sample sizes (3.7 ± 0.23 mg and 23.3 ± 0.75 mg). Each condition was performed at least on three different days, always in triplicate (Tables S2–S5). The workflow of the sample preparation, digestion, and analysis is illustrated in Figure 1.

MERX-T 40 mL certified graduated autosampler vials were used for acid digestion and analysis. Samples of the CRM IAEA-450 and BCR-414 were weighted directly inside the glass vials for the wet digestion procedure. Afterward, 3 mL of nitric acid (condition 1 and 2, Table S1) was added to each flask carefully. After 2–4 h of adding acid to each sample, vials were placed inside the oven at 85 °C for 12 h. Blank samples were prepared alongside each corresponding digestion condition following the same procedure as the CRM samples. After 12 h digestion, vials were left to cool down until room temperature and then spiked with 100 µL BrCl solution to oxidize all forms of mercury species into Hg(II). Samples were left overnight with BrCl before being used for analysis. Samples can be stable for at least 90 days.

### 2.3. Total Hg Analysis

Total mercury concentration was determined using a MERX^®^ Automated Total Mercury Analytical System (Brooks Rand Instruments, Seattle, WA, USA) consisting in MERX^®^ Autosampler, MERX Total Hg Purge & Trap Module, and a Brooks Rand Model III cold vapor atomic fluorescence spectroscopy (CVAFS) system. The procedures applied were based on manufacturer instructions and Method 1631 [22] published by the United States of America Environmental Protection Agency. Weighting was performed in two high-precision balances: CRM was weighted using a Quintix^®^ Semi-Micro Balance (Sartorius Quintix^®^, Göttingen, Germany), while all other samples were measured using a AT201 balance (Mettler-Toledo, Switzerland). Thermal acid digestions were carried out in Gallenkamp hotbox oven size 1 inside a chemical hood. Pyrolisation of glass vials used for digestion and Hg analysis were performed using a compact muffle furnace (Nabertherm, Germany).

Samples were analyzed following the EPA method 1631 guideline [22,23] and the MERX-T manufacture’s guidelines. Briefly, MERX-T 40 mL certified graduated autosampler vials were filled with 23 mL of ultrapure water for both samples and standard solution used for calibration. All glass vials filled with ultrapure water + Teflon caps were weighted to account for total mass (g) before adding digested sample aliquot. Samples were added to each flask and after weighted for gravimetric concentration determination. BrCl solution was added to each flask and left overnight to assure complete oxidation of mercury species. Next, the samples were sequentially reduced with NH_2_OH * HCl to destroy the free halogens and then reduced with SnCl_2_ to convert Hg(II) to volatile mercury (Hg^0^). The Hg^0^ was separated from the solution by purging with nitrogen. The Hg^0^ is collected onto a gold trap. The Hg is thermally desorbed from the gold trap into an inert gas stream that carries the released Hg^0^ to a second gold (analytical) trap. The Hg is desorbed from the analytical trap into a gas stream that carries the Hg into the cell of a CVAFS for detection.

### 2.4. Analytical Quality Assurance and Method Validation Parameters

Method validation is an important task of chemical analysis, and it is normally defined as if the analytical requirement and its verification towards the requirements for a specific intended use or application have been fulfilled. Following the IUPAC and Eurachem guidelines [26,27,28,29], single-laboratory validation of this method was undertaken to demonstrate that the method is fit-for-purpose. For that purpose, limit of detection (LOD), limit of quantitation (LOQ), linearity, working range, trueness, precision, and matrix comparison were evaluated.

LOD and LOQ are the most common parameters to be determined. However, it is important to be aware of the distinction between instrumental and method detection limits. LOD and LOQ of the method were determined using reagent blanks that go through the whole measurement procedure. Since blank corrections were performed to each sample’s final results per batch, changes were performed while calculating the standard deviation to determine LOD (Equation (1)) and LOQ (Equation (3)) as recommended and described in [29,30].

(1) $$LOD=3\times {S^{\prime }}_{0}$$

(2) $${S^{\prime }}_{0}=S_{0}\sqrt{\frac{1}{n}}+\frac{1}{nb}$$

(3) $$LOQ=10\times {S^{\prime }}_{0}$$

where *S*_0_ is the standard deviation of m single results at or near zero concentration; *S*′_0_ is the standard deviation used to determine LOD and LOQ, *n* is the number of replicates observations averaged; *nb* is the number of blank observations averaged when calculating the blank correction according to the measurement procedure.

Accuracy represents the closeness of a single result to a reference value and is usually associated with two components: trueness and precision and, more recently, uncertainty.

Precision of each measurement was assessed (Equation (4)) to evaluate the mean concentration of independent samples and it should not exceed 10% as described [31].

Trueness, providing how close the mean of an infinite number of results originated from the method is to the reference value (in our case, certified reference material), is usually expressed quantitatively as “bias”. On this validation method, the bias expressed as relative bias in per cent (Equation (5)) and the relative recovery in per cent (Equation (6)) were used to assess the trueness of the method among the different conditions tested as described in [29,30].

(4) $$RSD=\frac{\left(SD\right)}{\bar{x}}$$

(5) $$b\left(\%\right)=\frac{\begin{array}{ccc} \bar{x} & - & x_{ref} \end{array}}{x_{ref}}\times 100$$

(6) $$R\left(\%\right)=\frac{\bar{x}}{x_{ref}}\times 100$$

where *b*(%) stands for relative bias, *R*(%) stands for recovery in per cent, $\bar{x}$ is the mean of the measurement and *X_ref_* is the mean of the certified value.

For single laboratory validation, two conditions of precision determination are important [29]: 1. repeatability (Equation (7)), also called within-batch or within-run and refers to variations seen on replicate measurements made in one laboratory by a single analyst using the same equipment over a short time scale; 2. intermediate precision (Equations (8) and (9)) refers to a precision estimate obtained from replicate measurements made in a single laboratory under more variable conditions than repeatability conditions. A minimum of nine independent replicates performed in 4 different batches (Tables S2–S5) were used per condition to estimate intermediate precision, while for repeatability, a total *n* = 3 were used per condition/batch. The overall results are displayed in Table 1.

(7) $$S_{r}=\sqrt{MS_{w}}$$

(8) $$S_{b}=\sqrt{\frac{\begin{array}{ccc} MS_{b} & - & MS_{w} \end{array}}{n}}$$

(9) $$S_{I}=\sqrt{S_{r}^{2}+S_{b}^{2}}$$

where S*_r_* corresponds to repeatability standard deviation, *MS_w_* is the mean square within group variance; *MS_b_* is the mean square between group variance; *S_b_* corresponds to the total variation from the grouping factor; *S_I_* corresponds to intermediate precision.

All statistical analyses were performed at the 95% confidence level using Statistica software (version 13, TIBCO Software Inc., Palo Alto, CA, United States). Significant differences (*p* < 0.05) between the compiled bias results of condition 1 and condition 2 were evaluated using a *t*-test. Significant differences between conditions parameters were evaluated using the parametric one-way and two-way ANOVA (analysis of variance). All data analyses were tested for normality using the Kolmogorov–Smirnov test and for homoscedasticity using Leven’s test. A Tukey honestly significant difference (HSD) multiple comparison test was performed when significant differences were detected.

## 3. Results and Discussion

The total Hg content of plankton samples was determined using two decomposition conditions (Table S1). These conditions were selected considering a pre-screen of a wide range of conditions tested (Figure S1) where the three most important parameters were varied: digestion time, sample size, and type of oxidant concentration. Under two selected conditions differing only in acid concentration (50% *v*/*v* HNO_3_ vs. 65% *w*/*w* HNO_3_), a good precision (RSD < 10%) was found in the tested results (Tables S6–S9).

Following condition 1 (50% *v*/*v* HNO_3_), a method LOD of 0.0016 ng g^−1^ and LOQ of 0.0049 ng g^−1^ were determined, while condition 2 (65% *w*/*w* HNO_3_) presented a method LOD of 0.0035 ng g^−1^ and LOQ of 0.0107 ng g^−1^ as described in Equations (1) and (3). Taking into consideration the LOQ obtained, we estimated the range value of total mercury required to be present in plankton samples to be quantifiable using the proposed method (Table S10). Nevertheless, it is important to take these values as indicative and not definitive since variability along runs might occur. The working range was determined to obtain the interval over which the method provides results with an acceptable uncertainty. The method working range in the current method was not addressed since CRMs were used instead. However, we can define the lower part of the method range as the LOQ while the maximum not defined but should be related to the instrumental one. The instrumental linear working range of MERX-T is between 0.08 and400 ng/L. The linearity was evaluated between 5 and 2500 pg of the analyte mass. In this range, a total of six to nine calibration standard concentrations were used to build the calibration curves. Each standard was analyzed at least twice per batch. Linearity was evaluated by visual inspection of calibration and residual chart (Figure S2). All calibrations curves were plotted and displayed a 99% confidence level (R^2^ > 0.99). All values accepted passed the quality control acceptance criteria defined by US EPA method 1631.

**Figure 2:**
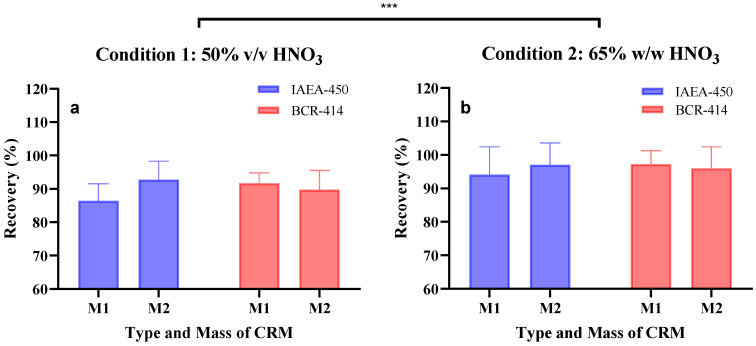
Recovery efficiency in percentage of (**a**) condition 1 and (**b**) condition 2 on the different conditions: CRM type: IAEA-450 and BCR-414, and different biomass: M1—3.1 ± 0.6 mg and M2—21.6 ± 1.5 mg. The error bar shows the standard deviation of the recovery values at different conditions. A two-way ANOVA was performed on the recoveries obtained between the digestion conditions and type of CRM used, with the two conditions tested being significantly different (*** *p* < 0.0005).

The relative bias (%) and relative recovery (%) obtained per run are described in Table S6–S9 of both conditions on two types of CRM (plankton material vs. unicellular alga) and two types of mass (3.7 ± 1.2 mg and 23.3 ± 3.0 mg). The overall results are presented in Table 1 and Figure 2. For condition 1, the relative biases ranged between −7.3% and −13.6% and were significantly different from condition 2 (*p* < 0.05, *t*-test), ranging between −2.8% and −5.9%. These results indicated that condition 2 is likely more fit-for-purpose than condition 1. Indeed, condition 2 presented better recovery efficiencies (94–97%) than condition 1 (86.0–93.0%). This was supported by subjecting both condition results to a statistical analysis using Two-way ANOVA with significant results (*p* < 0.0005). Furthermore, no statistical differences were found when comparing the CRM type vs. sample size within each condition (ANOVA test followed by Tukey’s post hoc test).

These results indicate that by using 65% *w*/*w* HNO_3_ for digestion of plankton samples within the low mass range, the determination of Hg should display a reliable outcome. To determine if the procedure would be precise and accurate over time, different runs were performed during a short period of time. With this information, we were able to determine the repeatability (*S_r_*) and the intermediate precision (*S_I_*) of both conditions (1 and 2). Results indicated a repeatability level < 7.9% for condition 2 and a variation of 4.8–11.9% for condition 1. Intermediate precision was equal to the repeatability results and, therefore, not displayed on the table. Considering all results obtained, condition 2 is more appropriate to be used. Additionally, no significant differences (ANOVA, *p* < 0.05) were found for each condition when comparing the accuracy results (Tables S6–S9) of each batch, which indicate that the method accuracy is likely constant over time.

The biomass of samples is a crucial factor when decomposing matrices to analyze of a specific analyte since low amounts of matrices can increase sample heterogeneity, and decrease method detection limits. Often, studies working with plankton communities generate low biomass, e.g., phytoplankton [32] or zooplankton [33], which poses challenges to obtain good recoveries results. Nevertheless, planktonic samples, e.g., Hg bioaccumulation assays of phytoplankton species in the laboratory range between 1.0–5.0 mg of dry weight (unpublished data). Few works on the digestion of planktonic samples for later trace elemental analysis have been published [11,16,21]. For instance, Taylor, Jackson and Chen [16] proposed a method for the determination of mercury speciation and trace elements in low biomass biological samples where different CRMs and environmental samples were used. By using BCR-414 (ranging between 20 and 50 mg) they were able to achieve a recovery of 94.9% which is within our results (94–97%). In our method, we were able to decrease the amount of sample size (range 2–20 mg) and maintain similar recoveries with low bias. Furthermore, we inferred the precision of our method when applying it to planktonic environmental samples. We used our method procedure (condition 2) in duplicate samples of plankton collected from surface water of Leman Lake (Switzerland) with a precision of 6% achieved (Table S11).

Lastly, when digesting and subsequently analyzing samples, a standard procedure is to use certified reference material as quality control for the recovery efficiency of the digestion method. However, it is important to always verify if by varying the amount of CRM vs. samples mass, the accuracy of the analysis is maintained within a good range. For example, as seen in Figure S1, when applying the proposed conditions (condition 1 and 2) with the recommended CRM mass (0.20 g), low recoveries were obtained in comparison with the other CRM mass (0.02 and 0.002 g), and therefore increasing the risk of bias.

## 4. Conclusions

Reliable methodologies for quantifying the total Hg content in plankton are necessary, since primary producers represent an important entry point for Hg in the aquatic food webs. We proposed a simple wet digestion procedure adapted for low biomass samples. A sensitive and accurate analytical method for validating total Hg targeting biological matrices with low mass sizes was developed and validated according to the IUPAC and Eurachem Guidelines. The recovery results of different CRMs indicate that nitric acid (65% *w*/*w* HNO_3_) at 85 °C for 12 h was the most efficient condition with good accuracy and precision recoveries (Recovery > 94%, RSD ≤ 10%). This procedure shows that efficient digestion of low biomass of planktonic samples followed by CVAFS analysis can be achieved with minimal losses and a simple digestion procedure.

## Figures and Tables

**Table 1:** Results of the analysis of the reference materials under the two procedures. Results describe the compilation of the different batches performed with recovery, precision, bias, and repeatability % of each condition.

Condition	CRM	Weight CRM (g)			Recovery (%)			BIAS (%)	Repeatability
		Mean ± SD			Mean	SD_pooled_	RSD_pooled_		*S_r_*
1.	IAEA-450	**M1** 0.0031	±	0.0008	86.4%	5.9%	6.8%	−13.6%	5.9%
		**M2** 0.0195	±	0.0010	92.8%	6.0%	6.4%	−7.3%	11.9%
	BCR-414	**M1** 0.0028	±	0.0008	91.7%	3.4%	3.7%	−8.5%	4.8%
		**M2** 0.0216	±	0.0034	89.8%	4.8%	5.4%	−10.5%	4.8%
2.	IAEA-450	**M1** 0.0026	±	0.0002	94.1%	7.6%	8.1%	−6.2%	7.9%
		**M2** 0.0231	±	0.0042	97.1%	7.5%	7.7%	−3.2%	7.7%
	BCR-414	**M1** 0.0041	±	0.0008	97.2%	4.6%	4.7%	−2.9%	4.6%
		**M2** 0.0221	±	0.0048	96.0%	6.9%	7.2%	−4.5%	6.9%

## Data Availability

The data presented in this study are available on request from the corresponding authors.

## Supplementary Materials

The following supporting information can be downloaded at: https://www.mdpi.com/article/10.3390/mps5020029/s1, Table S1: Description of digestion conditions applied at an initial phase to identify best conditions for the development and optimization of the procedure to obtain high recovery efficiency; Table S2: Digestion conditions and amounts of certified reference material IAEA-450 and BCR-414 and nitric acid (50% *v*/*v* HNO_3_ or 65% *w*/*w* HNO_3_) used on the first digestion batch; Table S3: Digestion conditions and amounts of certified reference material IAEA-450 and BCR-414 and nitric acid (50% *v*/*v* HNO_3_ or 65% *w*/*w* HNO_3_) used on the second digestion batch; Table S4: Digestion conditions and of the amounts of certified reference material IAEA-450 and BCR-414 and nitric acid (50% *v*/*v* HNO_3_ or 65% *w*/*w* HNO_3_) used on the third digestion batch. Na refers to any sample that did not pass the acceptance criteria of the analytical procedure; Table S5: Digestion conditions and amounts of certified reference material IAEA-450 and BCR-414 and nitric acid (50% *v*/*v* HNO_3_ or 65% *w*/*w* HNO_3_) used on the fourth digestion batch; Table S6: Results of digestion batch number 1 over 4. Information about CRM mass in each condition and its respective certified and measured concentrations. Additionally, accuracy, precision and recovery percentages are displayed; Table S7: Results of digestion batch number 2 over 4. Information about CRM mass in each condition and its respective certified and measured contents. Additionally, accuracy, precision and recovery percentages are displayed; Table S8: Results of digestion batch number 3 over 4. Information about CRM mass in each condition and its respective certified and measured concentrations. Additionally, accuracy, precision and recovery percentages are displayed; Table S9: Digestion batch number 4 over 4. Information about CRM mass in each condition and its respective certified and measured concentrations. Additionally, accuracy, precision and recovery percentages are displayed; Table S10: Estimation of the minimum amount of total mercury necessary to be present in 2 mg and 20 mg sample considered to the LOQ generated from the different conditions and the dilution (three times) performed for the analysis in triplicate. Results are expressed in ng of mercury per gram of sample; Table S11: Total mercury concentration of planktonic sample from Leman Lake (*n* = 2). Figure S1: Recovery efficiency results from the initial screening performed with different digestion conditions (Condition 1 and 6: 50% *v*/*v* HNO_3_ without ultrasound (WTU); Condition 2: 65% HNO_3_ WTU; Condition 3 and 5: 50% *v*/*v* HNO_3_ with ultrasound (WU); Condition 4: 65% HNO_3_ WU). More details about the different conditions are described in Table S1. Different dots correspond to different replicates measured. Digestion and replicates were performed along different runs; Figure S2: Calibration and residual plots of each run analysis.
