# Supplementary material for: Simple Acid Digestion Procedure for the Determination of Total Mercury in Plankton by Cold Vapor Atomic Fluorescence Spectroscopy
Source: Methods Protoc. 2022 Mar 25;5(2):29. doi: 10.3390/mps5020029 (PMC9029141; doi:10.3390/mps5020029)
Supplement: Supplementary file 1 [file mps-05-00029-s001.zip › mps-1624226-supplementary.pdf]

**Simple acid digestion procedure for the determination of total mercury in plankton by cold vapor atomic fluorescence spectroscopy**

João P. Santos[1,*], Lirie Mehmeti[1] and Vera I. Slaveykova[1,*]

[1]Environmental biogeochemistry and ecotoxicology, Department F.-A. Forel for Environmental and Aquatic Sciences, School of Earth and Environment Sciences, Faculty of Sciences, University of Geneva, 1211 Geneva, Switzerland

[*]Corresponding authors: Joao.RodriguesPereiraSantos@unige.ch (J.P. Santos); vera.slaveykova@unige.ch (V.I. Slaveykova)

Vera I. Slaveykova ORCID: 0000-0002-8361-2509

João P. Santos ORCID: 0000-0002-1899-2226

# Supplementary Material

**Table S1.** Description of digestion conditions applied at an initial phase to identify best conditions for the development and optimization of the procedure to obtain high recovery efficiency.

| Condition | Nitric acid | Amount of nitric acid | Temperature | Time of digestion | Pre-Ultrasound |
|---|---|---|---|---|---|
| 1* | 50% v/v $HNO_3$ | 3 mL | 85°C | 12 hours | No |
| 2* | 65% $HNO_3$ | 3 mL | 85°C | 12 hours | No |
| 3 | 50% v/v $HNO_3$ | 3 mL | 85°C | 12 hours | 10 min. |
| 4 | 65% $HNO_3$ | 3 mL | 85°C | 12 hours | 10 min. |
| 5 | 50% v/v $HNO_3$ | 3 mL | 85°C | 8 hours | 10 min. |
| 6 | 50% v/v $HNO_3$ | 3 mL | 85°C | 8 hours | No |

*Conditions selected for the method optimization

**Table S2.** Digestion conditions and amounts of certified reference material IAEA-450 and BCR-414 and nitric acid (50% v/v $HNO_3$ or 65% w/w $HNO_3$) used on the first digestion batch.

| CRM | Condition/Acid concentration | Replicate | Weight of CRM (g) | Weight of acid (g) |
|---|---|---|---|---|
| **IAEA450** | Condition 1 / 50% v/v $HNO_3$ | R1 | 0.0032 | 3.71725 |
| | | R2 | 0.0022 | 3.79136 |
| | | R3 | 0.0038 | 3.76018 |
| | | R1 | 0.0196 | 3.81142 |
| | | R2 | 0.0204 | 3.78940 |
| | | R3 | 0.0185 | 3.69523 |
| | Condition 2 / 65% w/w $HNO_3$ | R1 | 0.0028 | 4.25916 |
| | | R2 | 0.0025 | 4.12656 |
| | | R3 | 0.0025 | 4.21956 |
| | | R1 | 0.0213 | 4.15093 |
| | | R2 | 0.0201 | 4.28112 |
| | | R3 | 0.0279 | 4.08583 |
| **BCR414** | Condition 1 / 50% v/v $HNO_3$ | R1 | 0.0028 | 3.78277 |
| | | R2 | 0.0035 | 3.79261 |
| | | R3 | 0.0020 | 3.75466 |
| | | R1 | 0.0204 | 3.75476 |
| | | R2 | 0.0189 | 3.78158 |
| | | R3 | 0.0254 | 3.79087 |
| | Condition 2 / 65% w/w $HNO_3$ | R1 | 0.0033 | 4.19161 |
| | | R2 | 0.0040 | 4.12320 |
| | | R3 | 0.0049 | 4.07007 |
| | | R1 | 0.0273 | 4.14747 |
| | | R2 | 0.0178 | 4.20749 |
| | | R3 | 0.0212 | 4.24887 |

**Table S3.** Digestion conditions and amounts of certified reference material IAEA-450 and BCR-414 and nitric acid (50% v/v $HNO_3$ or 65% w/w $HNO_3$) used on the second digestion batch.

| CRM | Condition/Acid concentration | Replicate | Weight of CRM (g) | Weight of acid (g) |
|---|---|---|---|---|
| **IAEA450** | Condition 1 / 50% v/v $HNO_3$ | R1 | 0.0042 | 3.75038 |
| | | R2 | 0.0024 | 3.73934 |
| | | R3 | 0.0034 | 3.48495 |
| | | R1 | 0.0242 | 3.69380 |
| | | R2 | 0.0261 | 3.60498 |
| | | R3 | 0.0273 | 3.78008 |
| | Condition 2 / 65% w/w $HNO_3$ | R1 | 0.0031 | 4.18116 |
| | | R2 | 0.0060 | 4.04270 |
| | | R3 | 0.0046 | 4.15616 |
| | | R1 | 0.0229 | 4.18374 |
| | | R2 | 0.0237 | 4.15771 |
| | | R3 | 0.0242 | 4.21530 |
| **BCR414** | Condition 1 / 50% v/v $HNO_3$ | R1 | 0.0035 | 3.42441 |
| | | R2 | 0.0048 | 3.75815 |
| | | R3 | 0.0053 | 3.73949 |
| | | R1 | 0.0287 | 3.76293 |
| | | R2 | 0.0220 | 3.73817 |
| | | R3 | 0.0212 | 3.67177 |
| | Condition 2 / 65% w/w $HNO_3$ | R1 | 0.0031 | 4.07674 |
| | | R2 | 0.0037 | 4.14168 |
| | | R3 | 0.0047 | 4.08100 |
| | | R1 | 0.0216 | 4.23301 |
| | | R2 | 0.0275 | 4.21778 |
| | | R3 | 0.0255 | 4.14943 |

**Table S4.** Digestion conditions and of the amounts of certified reference material IAEA-450 and BCR-414 and nitric acid (50% v/v $HNO_3$ or 65% w/w $HNO_3$) used on the third digestion batch. Na refers to any sample that did not pass the acceptance criteria of the analytical procedure.

| CRM | Condition/Acid concentration | Replicate | Weight of CRM (g) | Weight of acid (g) |
|---|---|---|---|---|
| **IAEA450** | Condition 1 / 50% v/v $HNO_3$ | R1 | 0.0233 | 3.77754 |
| | | R2 | 0.0212 | 3.76495 |
| | | R3 | 0.0219 | 3.76947 |
| | Condition 2 / 65% w/w $HNO_3$ | R1 | 0.0022 | 4.09883 |
| | | R2 | 0.003 | 4.17654 |
| | | R3 | NA | NA |
| **BCR414** | Condition 2 / 65% w/w $HNO_3$ | R1 | 0.0054 | 3.98134 |
| | | R2 | 0.003 | 3.89726 |
| | | R3 | 0.0029 | 4.2136 |
| | | R1 | 0.0214 | 4.21056 |
| | | R2 | 0.0279 | 4.06167 |
| | | R3 | 0.0238 | 4.07188 |

**Table S5.** Digestion conditions and amounts of certified reference material IAEA-450 and BCR-414 and nitric acid (50% v/v $HNO_3$ or 65% w/w $HNO_3$) used on the fourth digestion batch.

| CRM | Condition/Acid concentration | Replicate | Weight of CRM (g) | Weight of acid (g) |
|---|---|---|---|---|
| **IAEA450** | Condition 1 / 50% v/v $HNO_3$ | R1 | 0.0025 | 3.79083 |
| | | R2 | 0.0022 | 3.80043 |
| | | R3 | 0.0027 | 3.83353 |
| | | R1 | 0.0215 | 3.24528 |
| | | R2 | 0.0254 | 3.5572 |
| | | R3 | 0.0220 | 3.47809 |
| | Condition 2 / 65% w/w $HNO_3$ | R1 | 0.0024 | 4.14458 |
| | | R2 | 0.0023 | 4.11394 |
| | | R3 | 0.0028 | 3.39231 |
| | | R1 | 0.0247 | 4.19323 |
| | | R2 | 0.0213 | 4.21024 |
| | | R3 | 0.0196 | 4.21588 |
| **BCR414** | Condition 1 / 50% v/v $HNO_3$ | R1 | 0.0027 | 3.79581 |
| | | R2 | 0.0024 | 3.54409 |
| | | R3 | 0.0021 | 3.66467 |
| | | R1 | 0.0272 | 3.59568 |
| | | R2 | 0.0192 | 3.55636 |
| | | R3 | 0.0198 | 3.29032 |

**Table S6.** Results of digestion batch number 1 over 4. Information about CRM mass in each condition and its respective certified and measured concentrations. Additionally, accuracy, precision and recovery percentages are displayed.

| CRM | Condition / Acid concentration | CRM mass [a] (g) | | | Certified content [a] (pg) | | | Measured content [a] (pg) | | | Accuracy Bias (%) | Recovery [a] (%) | | | Precision RSD [a] (%) |
|---|---|---|---|---|---|---|---|---|---|---|---|---|---|---|---|
| IAEA-450 | Condition 1 / | 0.0031 | ± | 0.0008 | 318.9 | ± | 84.1 | 278.5 | ± | 87.2 | -12.7% | 86.4% | ± | 6.0% | 6.9% |
| | 50% v/v $HNO_3$ | 0.0195 | ± | 0.0010 | 2028.0 | ± | 99.2 | 1839.5 | ± | 125.2 | -9.3% | 90.9% | ± | 9.2% | 10.2% |
| | Condition 2 / | 0.0026 | ± | 0.0002 | 270.4 | ± | 18.0 | 256.4 | ± | 32.0 | -5.2% | 95.1% | ± | 13.4% | 14.1% |
| | 65% w/w $HNO_3$ | 0.0231 | ± | 0.0042 | 2402.4 | ± | 436.8 | 2413.1 | ± | 745.0 | 0.4% | 99.1% | ± | 12.2% | 12.3% |
| BCR-414 | Condition 1 / | 0.0028 | ± | 0.0008 | 763.6 | ± | 207.2 | 686.7 | ± | 170.9 | -10.1% | 90.3% | ± | 4.9% | 5.4% |
| | 50% v/v $HNO_3$ | 0.0216 | ± | 0.0034 | 5952.4 | ± | 939.3 | 5614.3 | ± | 661.9 | -5.7% | 94.8% | ± | 8.0% | 8.5% |
| | Condition 2 / | 0.0041 | ± | 0.0008 | 1122.4 | ± | 221.4 | 1086.0 | ± | 203.8 | -3.2% | 97.0% | ± | 7.1% | 7.4% |
| | 65% w/w $HNO_3$ | 0.0221 | ± | 0.0048 | 6105.1 | ± | 1321.1 | 5984.9 | ± | 877.7 | -2.0% | 99.3% | ± | 11.1% | 11.2% |

[a] Mean ± SD (n=3): triplicate analysis of each Condition and CRM mass.

**Table S7.** Results of digestion batch number 2 over 4. Information about CRM mass in each condition and its respective certified and measured contents. Additionally, accuracy, precision and recovery percentages are displayed.

| CRM | Condition / Acid concentration | CRM mass [a] (g) | | | Certified content [a] (pg) | | | Measured content [a] (pg) | | | Accuracy Bias (%) | Recovery [a] (%) | | | Precision RSD [a] (%) |
|---|---|---|---|---|---|---|---|---|---|---|---|---|---|---|---|
| IAEA-450 | Condition 1 / 50% v/v $HNO_3$ | 0.0033 | ± | 0.0009 | 346.7 | ± | 93.8 | 299.9 | ± | 80.8 | -13.5% | 86.5% | ± | 6.9% | 7.9% |
| | | 0.0259 | ± | 0.0016 | 2690.1 | ± | 162.6 | 2830.5 | ± | 408.1 | -6.0% | 93.9% | ± | 4.1% | 4.4% |
| | Condition 2 / 65% w/w $HNO_3$ | 0.0046 | ± | 0.0015 | 474.9 | ± | 150.8 | 420.0 | ± | 129.1 | -11.6% | 88.7% | ± | 1.3% | 1.5% |
| | | 0.0236 | ± | 0.0007 | 2454.4 | ± | 68.2 | 2404.5 | ± | 143.4 | -2.0% | 97.9% | ± | 3.2% | 3.3% |
| BCR-414 | Condition 1 / 50% v/v $HNO_3$ | 0.0045 | ± | 0.0009 | 1251.2 | ± | 256.4 | 1150.7 | ± | 244.5 | -8.0% | 91.9% | ± | 0.9% | 1.0% |
| | | 0.0240 | ± | 0.0041 | 6614.8 | ± | 1136.7 | 5866.1 | ± | 1029.9 | -11.3% | 88.7% | ± | 1.9% | 2.1% |
| | Condition 2 / 65% w/w $HNO_3$ | 0.0038 | ± | 0.0008 | 1058.0 | ± | 223.1 | 1018.6 | ± | 191.1 | -3.7% | 96.6% | ± | 2.2% | 2.3% |
| | | 0.0249 | ± | 0.0030 | 6863.2 | ± | 828.2 | 6464.8 | ± | 832.6 | -5.8% | 94.1% | ± | 0.8% | 0.9% |

[a] Mean ± SD (n=3): triplicate analysis of each Condition and CRM mass.

**Table S8.** Results of digestion batch number 3 over 4. Information about CRM mass in each condition and its respective certified and measured concentrations. Additionally, accuracy, precision and recovery percentages are displayed.

| CRM | Condition / Acid concentration | CRM mass [a] (g) | Certified content [a] (pg) | Measured content [a] (pg) | Accuracy Bias (%) | Recovery [a] (%) | Precision RSD (%) |
|---|---|---|---|---|---|---|---|
| IAEA-450 | Condition 1 / 50% v/v $HNO_3$ | 0.0221 ± 0.0011 | 2301.9 ± 111.2 | 2182.9 ± 116.0 | -5.2% | 94.9% ± 6.1% | 6.4% |
| | Condition 2 / 65% w/w $HNO_3$ | 0.0030 ± 0.0008 | 312.0 ± 58.8 | 713.6 ± 40.8 | -6.4% | 94.2% ± 5.4% | 5.8% |
| BCR-414 | Condition 2 / | 0.0038 ± 0.0014 | 1039.6 ± 390.6 | 1021.7 ± 388.5 | -1.7% | 98.2% ± 2.5% | 2.6% |
| | 65% w/w $HNO_3$ | 0.0244 ± 0.0033 | 6725.2 ± 907.2 | 6339.5 ± 735.7 | -5.7% | 94.5% ± 4.2% | 4.5% |

[a] Mean ± SD (n=3): triplicate analysis of each Condition and CRM mass.

**Table S9.** Digestion batch number 4 over 4. Information about CRM mass in each condition and its respective certified and measured concentrations. Additionally, accuracy, precision and recovery percentages are displayed.

| CRM | Condition / Acid concentration | CRM mass [a] (g) | | | Certified content [a] (pg) | | | Measured content [a] (pg) | | | Accuracy Bias (%) | Recovery [a] (%) | | | Precision RSD [a] (%) |
|---|---|---|---|---|---|---|---|---|---|---|---|---|---|---|---|
| IAEA-450 | Condition 1 / 50% v/v $HNO_3$ | 0.0025 | ± | 0.0003 | 256.5 | ± | 26.2 | 221.5 | ± | 28.7 | -13.6% | 86.3% | ± | 4.7% | 5.5% |
| | | 0.0230 | ± | 0.0021 | 2388.5 | ± | 220.7 | 2184.6 | ± | 233.0 | -19.9% | 91.4% | ± | 1.6% | 1.8% |
| | Condition 2 / 65% w/w $HNO_3$ | 0.0025 | ± | 0.0003 | 260.0 | ± | 27.5 | 256.0 | ± | 29.2 | -1.5% | 98.5% | ± | 4.7% | 4.7% |
| | | 0.0219 | ± | 0.0026 | 2274.1 | ± | 270.1 | 2093.8 | ± | 229.9 | -7.9% | 94.3% | ± | 4.5% | 4.7% |
| BCR-414 | Condition 1 / 50% v/v $HNO_3$ | 0.0024 | ± | 0.0003 | 665.2 | ± | 78.7 | 616.9 | ± | 72.4 | -7.3% | 92.8% | ± | 3.2% | 3.4% |
| | | 0.0221 | ± | 0.0045 | 6090.4 | ± | 1229.8 | 5211.6 | ± | 962.0 | -14.4% | 85.8% | ± | 1.4% | 1.6% |

[a] Mean ± SD (n=3): triplicate analysis of each Condition and CRM mass.

**Table S10.** Estimation of the minimum amount of total mercury necessary to be present in 2 mg and 20 mg sample considered to the LOQ generated from the different conditions and the dilution (three times) performed for the analysis in triplicate. Results are expressed in ng of mercury per gram of sample.

| **Amount of total Hg required per sample (ng $g^{-1}$)** | | | |
|---|---|---|---|
| **Condition 1 /50% v/v HNO3** | | **Condition 2 /65% w/w HNO3** | |
| LOQ: 0.0049 ng $g^{-1}$ | | LOQ: 0.0107 $ng^{-1}$ | |
| 2 mg of sample | 20 mg of sample | 2 mg of sample | 20 mg of sample |
| 7.4 | 0.7 | 16.0 | 1.6 |

**Table S11.** Total mercury concentration of planktonic sample from Leman Lake (n=2).

| **Planktonic sample from Leman Lake (pg Hg $mg^{-1}$ dry weight)** | | | |
|---|---|---|---|
| **Replicates** | **Average** | **SD** | **RSD** |
| 10.6<br>9.7 | 10.1 | 0.6 | 6% |

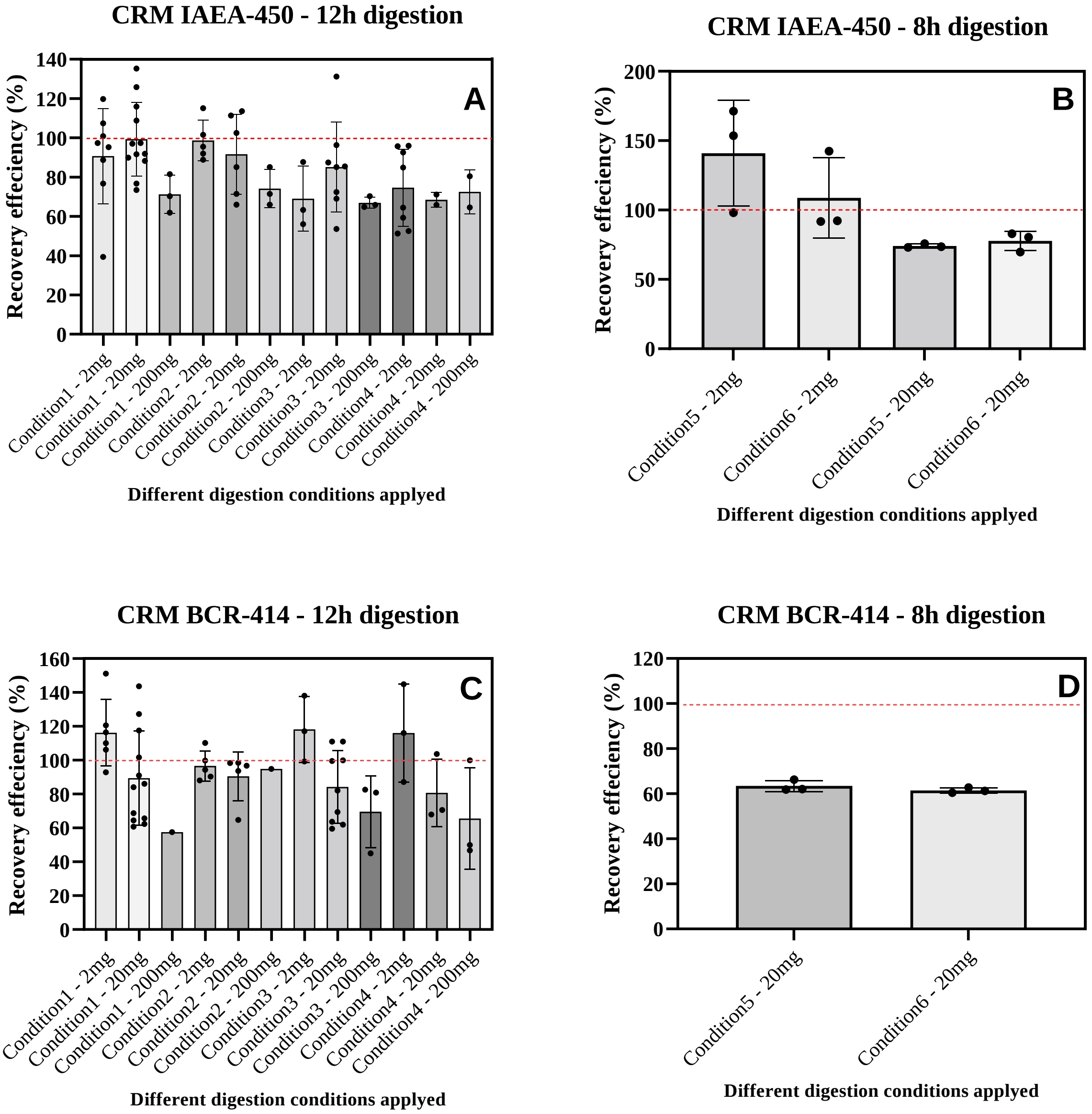


**Figure S1.** Recovery efficiency results from the initial screening performed with different digestion conditions (Condition 1 and 6: 50% v/v $HNO_3$ without ultrasound (WTU); Condition 2: 65% $HNO_3$ WTU; Condition 3 and 5: 50% v/v $HNO_3$ with ultrasound (WU); Condition 4: 65% $HNO_3$ WU). More details about the different conditions are described in Table S1. Different dots correspond to different replicates measured. Digestion and replicates were performed along different runs.

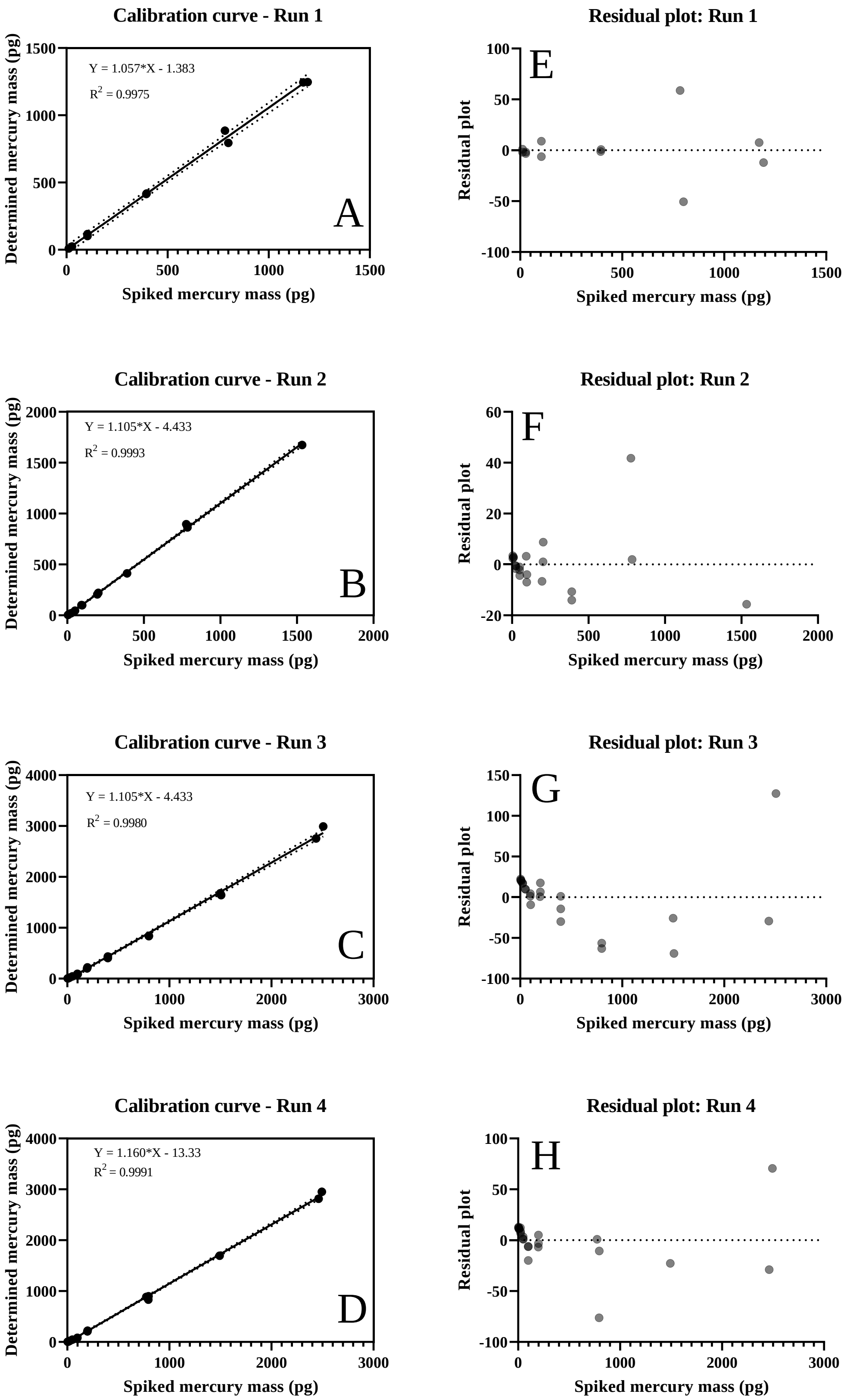


**Figure S2.** Calibration and residual plots of each run analysis.
